# Epidemiological Profile of Human Papillomavirus in a Healthcare Center in Portugal: Implications for Public Health Policies

**DOI:** 10.7759/cureus.57023

**Published:** 2024-03-27

**Authors:** António Luz Pereira, Deolinda Chaves Beça, Maria Buchner Sousa, Margarida Vaz Pinto, Daniela Bento, Inês Leal, Mariana Bandeira

**Affiliations:** 1 Unidade de Saúde Familiar (USF) Prelada, Unidade Local de Saúde (ULS) Santo António, Porto, PRT; 2 Unidade de Saúde Familiar (USF) Carvalhido, Unidade Local de Saúde (ULS) Santo António, Porto, PRT; 3 Cuidados de Saúde Primários (CSP) Viseu Dão-Lafões, Unidade Local de Saúde (ULS) Viseu Dão-Lafões, Viseu, PRT

**Keywords:** hpv genotypes, uterine cervical cancer, pap smear study in cancer cervix, cervical cancer screening, human papillomavirus (hpv)

## Abstract

Introduction

Human papillomavirus (HPV) infection, a prevalent sexually transmitted disease, affects the majority of sexually active individuals at least once in their lifetime. Cervical cancer stands as a significant contributor to mortality among women. Cervical cancer screening (CCS) and HPV vaccination are recent, with few studies about their impact on the prevalence of HPV types. The emergence of novel predominant pathogen strains can be driven by vaccine-induced pathogen strain replacement, thereby enhancing and altering selection.

Objective

The aim of the study was to characterize the high-risk (HR) HPV infection in two Portuguese primary care units (PCUs).

Materials and methods

In this observational, cross-sectional, and descriptive study, we included women aged 25-64 years and registered in two PCUs, who were screened by SiiMA Rastreios (population-based screening management application), and were HR-HPV positive, between August 2015 and May 2018. The results of cervical cancer screening (CCS) can be accessed through the SiiMA Rastreios information system. For data treatment, we used MS Excel (Microsoft Corporation, Redmond, Washington, USA), IBM SPSS Statistics for Windows, Version 20 (Released 2011; IBM Corp., Armonk, New York, USA), and non-parametric tests.

Results

In our study, we included 4,614 women aged between 25 and 64 years old. CCS was performed on 24.47%, of whom 39.95% were tested for HR-HPV. The infection rate was 18.85%, and all 14 types of infection were identified. The most common HPV type was 31, followed by 16 and 68. We found HPV other than 16/18 in 84.43%. We found coinfections in 34.1%, with no statistically significant difference by age group. In the 25-34 age group, the incidence of infection was 33.7% vs. 17.54% in the 35-54 age group and 4.55% in the 55-64 age group. HPV16 was the most common infection in the 25-34 age group. In nulliparous women, the most common was HPV31. The relationship between smoking habits and HR-HPV infection was statistically significant, but economic insufficiency was not.

Conclusion

The infection incidence in this study was slightly higher than in the 2011 national study. Statistically, the infection rate was significantly higher in the younger age groups. The most frequent type varied from the national and international study results. This may be due to regional differences in HPV infection, changes in the pattern of incidence, or the effect of vaccination. The HPV pattern may be changing, so the scientific community must keep updated to develop increasingly efficient screening and vaccination programs.

## Introduction

Human papillomavirus infection (HPV) is one of the most common sexually transmitted diseases, such that most sexually active people have contact with the virus at least once in their lifetime [1-4].

More than 170 types have been identified [5], and about 14 are associated with increased risk of cancer, also known as high-risk (HR)-HPV 16, 18, 31, 33, 35, 39, 45, 51, 52, 56, 58, 59, 66, and 68 [1]. HPV 16 is the most common. HPV 16 and 18 together constitute the most oncogenic types, accounting for 70%-75% of cancerous lesions. HPV 16, 18, 31, 33, 45, 52, and 58 are responsible for an estimated 90% of cases worldwide [1,4,6].

HPV infections have a broad clinical spectrum and may correspond to asymptomatic or subclinical infections and anogenital or premalignant or malignant lesions, particularly cervical cancer. Nearly all cases of cervical cancer can be attributable to HPV infection [1].

Invasive cervical cancer is the second-leading cause of death in women under 44 years old [7]. Every year worldwide, around 570,000 cases of cervical cancer are diagnosed, and about 311,000 women die [1]. In Portugal, according to the 2010 “Registo Nacional Oncológico,” the incidence of cases is 8,9/100,000 [7].

Cervical cancer screening (CCS) is one of the oldest and most effective screening programs, with substantial decreases in mortality since its implementation. Although the increasing use of vaccination against high-risk human papillomavirus (HR-HPV) is expected to play a major role in the prevention of cervical cancer, screening will still be needed, at least for non-vaccinated women and high-risk groups [8].

Currently, in Portugal, CCS is recommended to be performed by nucleic acid-based HR-HPV testing every five years for women aged between 25 and 60 years [3,6]. Women registered at a primary care unit are invited to CCS through the platform SiiMA Rastreios [8,9].

In positive cases for HPV 16 and 18, the patients should be referred to the hospital for cervical pathology consultation [8]. For those testing positive for HR-HPV types other than 16 and 18, cytology should be performed. Patients with atypical squamous cells of undetermined significance (ASC-US) or abnormal cytology results should be referred to a cervical pathology consultation in a hospital. Patients with normal or negative cytology must repeat in one year [9].

Three vaccines have now been prequalified, all protecting against both HPV 16 and 18 [1]. In 2008, Portugal included the tetravalent vaccine against HPV in the national vaccination plan for 13-year-old girls. Between 2009 and 2011, a review of all young women up to 17 years old was carried out [4]. Since January 2017, the national vaccination plan has recommended the vaccination of 10-year-old girls with the nonavalent vaccine (6, 11, 16, 18 31, 33, 45, 52, 58). Since October 2020, the plan has included boys of the same age [10,11].

By enhancing and altering selection processes by protecting hosts against specific pathogen strains, vaccination has the potential to stimulate the emergence of novel predominant pathogen strains, a phenomenon known as vaccine-induced pathogen strain replacement [12,13].

CCS and HPV vaccination are relatively recent, with few studies of their effect on the prevalence of HPV types. Our study aimed to characterize the population of two Portuguese primary care units with respect to social and behavioral factors related to HR-HPV infection.

## Materials and methods

HPV Epidemiology and Risk Assessment (HERA) is an observational, cross-sectional, and descriptive study conducted in May 2018. The study included women with a medical registration in two primary care units in the north of mainland Portugal, namely Unidade de Saúde Familiar (USF) Prelada and USF Carvalhido, which serve densely populated urban areas near the coast.

Our study incorporated specific inclusion and exclusion criteria. Women within the age range of 25 to 64 years were considered for inclusion if they met the following criteria: eligibility for cervical cancer screening (CCS), initiation of sexual activity, absence of hysterectomy, no ongoing cervical cancer treatment, and the absence of gynecological signs or symptoms. Conversely, individuals were excluded from the study if they had a history of cervical abnormalities, had undergone hysterectomy, or expressed a refusal to participate in the screenings. We implemented these criteria to ensure a homogeneous and relevant participant cohort for the investigation.

Screening was performed by SiiMA Rastreios, the national software for screening cancer eligibility, with detected HR-HPV, between August 2015 and May 2018.

The results of CCS can be accessed through the SiiMA Rastreios information system, a population-based screening management application. 

For data treatment, we used statistical analysis software such as MS Excel (Microsoft Corporation, Redmond, Washington, USA) and IBM SPSS Statistics for Windows, Version 20 (Released 2011; IBM Corp., Armonk, New York, USA) and non-parametric tests. The results were deemed statistically significant when the p-value was less than 0.05.

## Results

A total of 4,614 women aged between 25 and 64 years were identified. Of those, 24.47% (n=1,129) received CCS, and of these, 39.95% (n=451) were tested for HR-HPV by SiiMA Rastreios. None of the women had been vaccinated against HPV.

The incidence of HR-HPV infection was 18.85% (n=85). All 14 HR types were identified. The types were led by HPV 31 (13.71%, n=17), followed by 16 (12.90%, n=16) and 68 (11.29%, n=14) (Table 1). Of the total patients, 84.68% (n=103) had HR-HPV types other than 16 or 18. Coinfections were shared by 34.1%, with no statistically significant difference by age group. There were no patients with HPV 16 and 18 who had coinfections (Table 2). 

**Table 1:** HR-HPV type distribution, cases (N), and corresponding percentages in the study participants. HR-HPV: high-risk human papillomavirus.

	HR-HPV type	N	%
1	31	17	13.71%
2	16	16	12.90%
3	68	14	11.29%
4	52	12	9.68%
5	66	12	9.68%
6	58	11	8.87%
7	59	9	7.26%
8	51	7	5.65%
9	39	6	4.84%
10	56	5	4.03%
11	33	4	3.23%
12	45	4	3.23%
13	18	3	2.42%
14	35	2	1.61%
15	Other than 16/18	2	1.61%
	Total	124	100%

**Table 2:** Co-infection HR-HPV type distribution, cases (n), and corresponding percentages in the study participants. HR-HPV: high-risk human papillomavirus.

Co-infection (no of types)	N	%	
1	54	63.53%	
2	20	23.53%	34.12%
3	7	8.24%	
4	2	2.35%	
Unknown (other than 16/18)	2	2.35%	
Total	85	100%	

The HPV incidence decreased with increasing age. In the age group 25-34 (n=34), the incidence of HPV-AR infection was 33.7% vs. 17.54% of those between 35 and 54 years old (p=0.007) and 4.55% between 55 and 64 years old (p=0.01). Between 25 and 34 years (n=34), HPV 16 was the most common (22.2%), followed by HPV 66 (15.6%).

In nulliparous women, the most common was HPV 31 (17.8%), followed by HPV 16 (12.3%), while in the others, the most common was HPV 68 (16.3%), followed by HPV 16 (14.3%). 

The relationship between smoking habits and HR-HPV infection was statistically significant (p=0.003), but economic insufficiency was not (p=0.791). Of women testing positive for HR-HPV, 12.4% (n=56) were referred for cervical pathology consultation in a hospital, of whom 73.2% (n=41) made an appointment, but 19.5% (n=8) missed the appointment.

## Discussion

The observed results underscore the importance of CCS and HPV testing in our study population. Despite a relatively low participation rate in CCS (24.47%), a noteworthy proportion (39.95%) of screened individuals underwent testing for HR-HPV.

The incidence of HR-HPV infection in this study (18.85%) was slightly higher than that in the national CLEOPATRA study in 2011 (14.84%), which considered users between 18 and 64 years old [14]. Statistically, the incidence of HR-HPV infection was significantly higher at younger ages (25-34) [1,14].

In our study, the most prevalent type, HPV 31, contrasts with findings in both national and international studies, where HPV 16 is commonly reported as the predominant type [14,15].

This departure from established patterns raises questions about the distribution and prevalence of specific HR-HPV types in our study population. Notably, a significant proportion (84.68%) of patients exhibited HR-HPV types other than 16 or 18, suggesting a diverse landscape of HR-HPV infections in the examined cohort. Furthermore, the age-stratified analysis revealed a decreasing trend in HPV incidence with increasing age, with distinct prevalence patterns observed across different age groups. Understanding these variations is crucial for tailoring screening and prevention strategies based on age-specific risk profiles.

Significantly, the association between smoking habits and HR-HPV infection demonstrated statistical significance (p=0.003), highlighting the potential role of lifestyle factors in influencing HR-HPV prevalence. This finding aligns with a corroborating study, underscoring the significance of the association between smoking habits and HR-HPV infection [16]. Conversely, economic insufficiency did not show a statistically significant association with HR-HPV infection (p=0.791), suggesting a more nuanced interplay of socio-economic factors in this context. Moreover, the referral of HR-HPV-positive women for cervical pathology consultation in a hospital revealed a notable rate of appointment attendance (73.2%), underscoring the importance of prompt follow-up in managing identified high-risk cases. However, the observed rate of missed appointments (19.5%) signals a potential area for targeted interventions to improve adherence to necessary clinical evaluations. Overall, these findings contribute valuable insights into the complex dynamics of HR-HPV prevalence, risk factors, and healthcare utilization within our study population.

In our study population, the quadrivalent vaccine could have prevented around 16% of the detected infections, whereas the nonavalent vaccine would have had an effect on around 55% of these infections [17]. Will it be necessary to change immunization strategies by taking into account more recent data? Should we have a passive attitude until the nonavalent vaccine, recently included in the PNV, has an impact on the prevalence of HR-HPV types? Should we revaccinate women vaccinated with quadrivalent? Would it be worthwhile to develop a vaccine with different types of the quadrivalent vaccine, as a complement to this one? If so, who should we vaccinate?

It is essential to acknowledge the limitations of our study, particularly regarding its application in only two Portuguese primary care units. This restricted scope implies caution in generalizing our findings to broader populations. The specificity of these healthcare settings may influence the prevalence and characteristics of HPV infections, potentially limiting the broader applicability of our results. Future research with a more extensive and diverse participant pool would be instrumental in enhancing the generalizability of our findings.

There are many issues to go deeper into, and it is important to invest in work in this area, trying to answer the pertinent questions. The HPV-type pattern may be changing [18], so the scientific community must keep updated to develop increasingly efficient HPV screening and vaccination programs.

## Conclusions

Our study contributes to the understanding of HPV epidemiology and its implications for vaccination and screening strategies. The slightly higher incidence of HR-HPV infection we observed in our study compared to national averages underscores the importance of ongoing surveillance and intervention efforts. Moreover, the significant differences in HPV incidence across age groups highlight the need for targeted prevention initiatives, particularly among younger populations. The discrepancy in HPV-type distribution, with HPV 31 being the most frequent type in our study, suggests potential regional variations or changes in infection patterns over time, possibly influenced by vaccination programs. Our findings also indicate that existing HPV vaccines could have prevented a substantial proportion of detected infections, albeit with varying effectiveness depending on the vaccine type. As the landscape of HPV vaccination evolves with the introduction of the nonavalent vaccine, there is a pressing need to reassess immunization strategies and consider the implications for revaccination and vaccine development. In future studies focusing on assessing the risk of HPV infections, it is important to consider factors such as the age of sexual debut and exposure to multiple sexual partners, as these have been significantly associated with HPV infection.

Continued research in this area is paramount to inform evidence-based public health interventions and ensure the effectiveness of HPV screening and vaccination programs in reducing the burden of HPV-related diseases. Moving forward, close collaboration among researchers, policymakers, and healthcare providers will be essential to address emerging challenges and optimize prevention efforts against HPV.
